# Opposing fates: a bipolar cellular model for FMDV replication shaped by ^12^C^6+^heavy-ion mutagenesis

**DOI:** 10.3389/fcimb.2025.1715061

**Published:** 2026-01-07

**Authors:** Xiangdong Song, Yan Cui, Fanglan An, Yajun Li, Jianping Liang, Shiyu Tao, Xuerong Liu

**Affiliations:** 1 College of Veterinary Medicine, Gansu Agricultural University, Lanzhou, Gansu, China; 2 China Agricultural Vet Biology and Technology Co., Ltd, Lanzhou, Gansu, China; 3 Institute of Modern Physics (IMP), Chinese Academy of Sciences, Lanzhou, Gansu, China; 4 Gansu Normal University for Nationalities, Hezuo, Gansu, China

**Keywords:** ^12^C^6+^ heavy-ion, BHK-21, transcription factor, FMDV, BHK-21 cell line

## Abstract

**Introduction:**

By pioneering the use of an 80 MeV/u ^12^C^6+^ heavy-ion beam for mutagenesis, we have engineered a stably polarized BHK-21 cell model for FMDV replication.

**Methods:**

This approach yielded two distinct clones: a highly antiviral line (BHK-5) and a highly proviral line (BHK-7). Multi-omics analyses were employed to investigate the mechanisms driving these divergent phenotypes.

**Results:**

The divergent phenotypes stem from a profound reprogramming of host transcriptional networks. The antiviral BHK-5 clone exhibits a pre-activated innate immune state, leveraging *RIG-I/TLR* signaling for a rapid interferon response and viral clearance via autophagy. In stark contrast, the proviral BHK-7 clone enhances glycolysis and activates the PI3K-Akt pathway to suppress *TNF*-mediated immunity and hijack the *G2/M* cell cycle phase, forming organized "virus factories." At the core of this reprogramming lies a systemic remodeling of transcription factor circuits, particularly within the Runt and *C2H2* zinc-finger families.

**Discussion:**

Our work demonstrates that ^12^C^6+^ heavy-ion mutagenesis can rewire the host immunity-metabolism-cell cycle axis to dictate infection outcomes, providing a powerful framework and cellular toolkit for developing high-yield vaccine substrates and novel antiviral strategies.

## Introduction

Foot-and-mouth disease (FMD), caused by the Foot-and-mouth disease virus (FMDV), is an acute, highly contagious viral infection that severely impacts cloven-hoofed animals, inflicting substantial economic losses on global livestock production and trade (King et al., 2024; Humphreys et al., 2025). Vaccination remains the most effective strategy for controlling FMD. Current inactivated FMD vaccines rely on large-scale viral propagation in cell culture, followed by inactivation and purification. Since the 1960s, the Baby Hamster Kidney (BHK-21) cell line has served as the primary substrate for FMD vaccine production, owing to its high permissiveness to FMDV and rapid growth kinetics. However, conventional adherent culture systems present major scale-up limitations, including operational complexity, high costs, and low space efficiency. Although adaptation to suspension culture has improved throughput (Park et al., 2021), inherent limitations of BHK-21 cells—such as suboptimal viral yields, passage-dependent instability, and variable susceptibility to field strains (Khoshnood et al., 2025)—continue to constrain vaccine yield and potency. To address these challenges, genetic enhancement of cell substrates via engineering strategies to develop lineages with improved viral productivity and manufacturability has become a critical technological focus.

Conventional mutagenesis techniques (e.g., chemical mutagens or UV/γ-irradiation) introduce genetic variation but exhibit nonspecific and dispersed energy deposition, typically resulting in simple DNA lesions. Consequently, they often fail to generate mutant lines with stably enhanced phenotypes. In contrast, heavy-ion beam irradiation has emerged as a potent physical mutagen, with distinct advantages in plant breeding and microbial engineering (Yanagisawa et al., 2022; Guo et al., 2024; Ishii et al., 2024). Unlike conventional radiations, heavy ions (e.g.,^12^C^6+^) exhibit high linear energy transfer (LET) and a defined penetration depth, generating dense ionization tracks that induce complex, clustered DNA damage—including double-strand breaks (DSBs) (Sutherland et al., 2000; Hagiwara et al., 2019; Mladenova et al., 2022a; Mladenova et al., 2022b). This damage profile facilitates pronounced genomic rearrangements and stable genetic variation, offering new opportunities to select cell lines with desirable traits.

Although ^12^C^6+^ heavy-ion irradiation is well established in cancer therapy and materials science, its systematic application in animal cell engineering, particularly for targeted modification of host cells to alter viral replication efficiency, remains underexplored. While heavy ions have been used in plant breeding (Guo et al., 2024; Ishii et al., 2024), their potential to reprogram entire regulatory networks in animal cells to achieve stable anti-viral or pro-viral phenotypes is a largely uncharted frontier. Given that viral replication is governed by multifaceted virus–host interactions involving innate immune responses, metabolic pathways, and cell-cycle control (Moreno-Altamirano et al., 2019; Camps et al., 2025),we hypothesized that the unique radiobiological properties of ^12^C^6+^ heavy-ion irradiation could be leveraged to induce profound heritable genomic alterations in BHK-21 cells and to isolate clonal populations exhibiting stable, opposing phenotypes—a highly pro-viral, pro-viral state versus a highly anti-viral, anti-viral one—toward FMDV replication. We further postulated that these phenotypes result from system-wide rewiring of host gene regulatory networks rather than from monogenic mutations.

In this study, we developed a screening and characterization pipeline based on ^12^C^6+^ heavy-ion irradiation to isolate BHK-21-derived clonal lines with polarized FMDV replication capacities. We employed integrated multi-omics approaches, including transcriptomics and computational network inference to systematically compare gene expression profiles, signaling pathways, and TF regulatory networks in these lines before and after viral infection. Complemented by ultrastructural analyses, this framework aimed to elucidate the molecular mechanisms underlying divergent virus–host interactions (Ma et al., 2025). This work is expected to yield candidate producer cell lines with enhanced performance for FMD vaccine production (Song et al., 2025a), while providing tractable models and conceptual insights into the complex regulatory networks governing FMDV–host dynamics.

## Materials and methods

### Cells and viruses

Wild-type BHK-21 cells (ATCC CCL-10), foot-and-mouth disease virus (FMDV) strain O/MYA98 was supplied by China Agricultural Vet Biology and Technology Co., Ltd.

### ^12^C^6+^ heavy-ion irradiation of BHK-21 cells

BHK-21 cells were plated in 35 mm dishes at 1.0×10^5^ cells/mL and irradiated, as we previously described (Song et al., 2025a; Song et al., 2025b), using a ^12^C^6+^ heavy ion beam at the Heavy Ion Research Facility in Lanzhou (HIRFL), Institute of Modern Physics, Chinese Academy of Sciences. The initial beam energy was 80.55 MeV/u.

### Screening and identification of monoclonal cell lines

**Figure 1:**
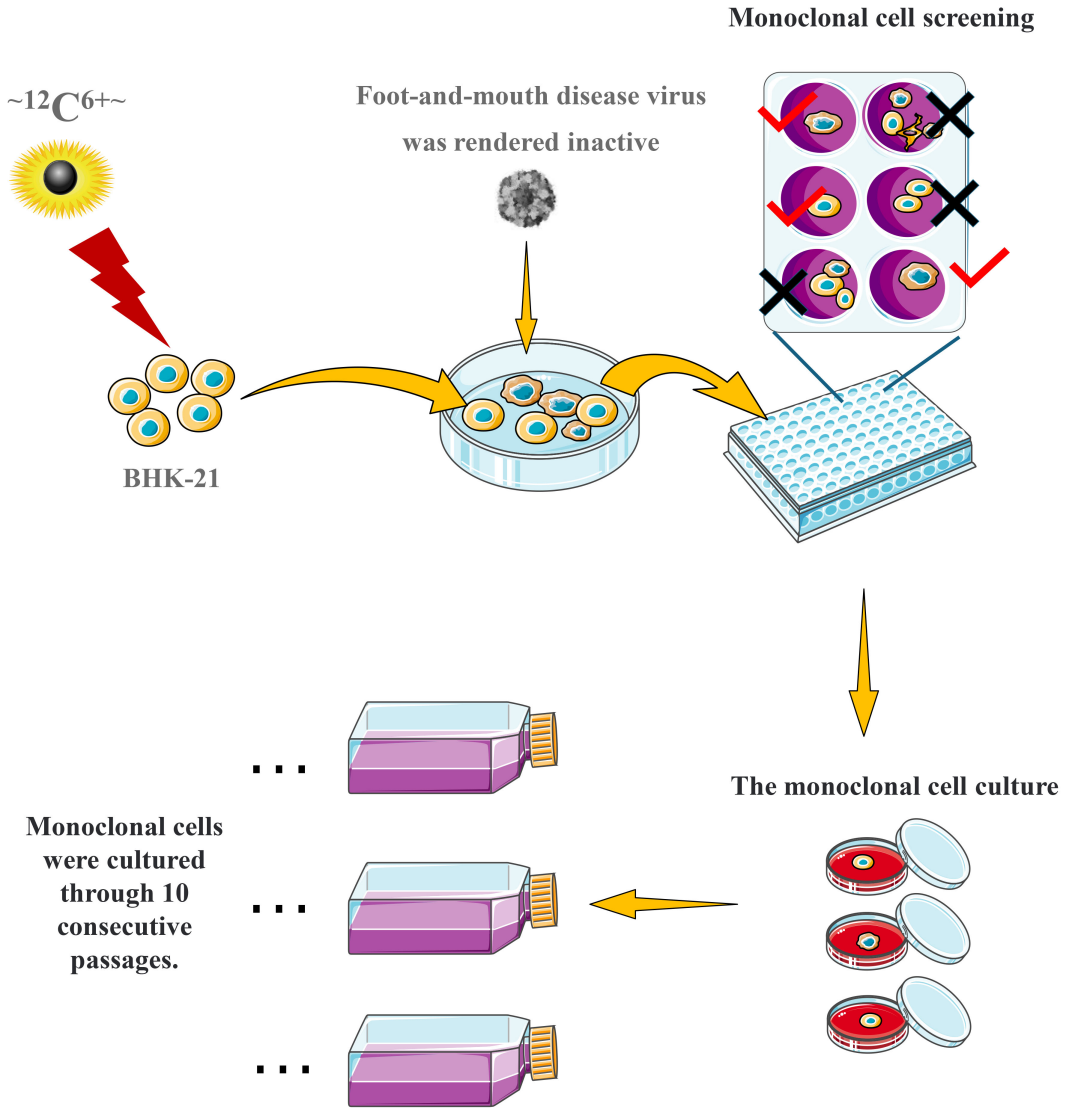
Schematic representation of the cell screening process.

BHK-21 cells (>95% viability) were seeded at 1.0×10^5^ cells/mL in 2.0 cm² dishes and irradiated (5.0, 10.0, or 15.0 Gy). After 48 h of culture in fresh medium, surviving cells were harvested and reseeded at the same density. Using limiting dilution in 96-well plates (**Figure 1**), single-cell clones were isolated and screened for morphological, proliferative, and growth differences from the parental line. Candidate clones underwent three rounds of subcloning to establish stable lines (passages T1-T3), which were then expanded from 96-well plates to 6-well plates and T25 flasks.

### Quantification of FMDV 146S particles and RNA analysis

Selected mutant and parental BHK-21 cell lines were seeded in 6-well plates at 5.0×10^5^ cells/mL. Upon adherence, cells were washed with PBS and inoculated with FMDV at an MOI of 0.1 for 1 h at 37 °C to allow adsorption. The inoculum was then replaced with DMEM (ThermoFisher Scientific C11995500BT) containing 2% FBS (Gibco 10099141C), and infection continued for 16 h. The viral supernatant was collected, adjusted to 5 mL with DMEM, and subjected to three freeze-thaw cycles to lyse cells prior to quantification of FMDV 146S particles.

### RT-qPCR analysis

Confluent monolayers of selected BHK-21 cell lines in 6-well plates were infected with FMDV (MOI = 0.1) for 1 h at 37 °C. After adsorption, the medium was replaced with DMEM containing 2% FBS for a 16 h infection period. Total RNA was extracted from the cells, reverse-transcribed into cDNA using the PrimeScript™ II kit (Takara Bio 6210A), and the product was used directly as the template for qPCR following the kit protocol (2×SYBR Green qPCR Mix. AH0104-B).

### TCID_50_ assay

Confluent monolayers of parental BHK-21 cells in 96-well plates were inoculated with tenfold serial dilutions of FMDV ranging from 10^-^¹ to 10^-9^. Following 1 h of virus adsorption at 37 °C, unbound viral particles were removed by washing with PBS, and the cells were maintained in DMEM supplemented with 2% FBS. After 16 h of incubation, cytopathic effects (CPE) were recorded. The viral titer, expressed as 50% tissue culture infectious dose (TCID_50_), was calculated using the Reed–Muench method. Each assay was performed in triplicate.

### Transcriptome sequencing and bioinformatic analysis

Total RNA was extracted using TRIzol (TRIzol™ Reagent 15596018) reagent from triplicate samples of uninfected and FMDV-infected (MOI = 0.1, 6 hpi) BHK-5, BHK-7, and parental BHK-21 cells. Following RNA integrity assessment, sequencing libraries were prepared and paired end sequenced on an Illumina NovaSeq 6000 platform. Clean reads were aligned to the golden hamster genome (GCF_017639785.1) using HISAT2. Gene expression was quantified as FPKM with feature Counts, and differential expression analysis (|log_2_FoldChange| > 1, adjusted *p*-value < 0.05) was performed using DESeq2.

### Transcription factor regulatory network analysis

For species included in the AnimalTFDB or PlantTFDB databases, TFs were annotated by querying these databases with Differentially Expressed Gene (DEG) gene identifiers. For species not covered by these resources, TF annotation was performed based on Pfam domain annotations, supplemented by family classification from the Transcription Factor Prediction Database (DBD). With reference to AnimalTFDB 3.0, TFs were identified among DEGs, and their expression patterns (upregulated or downregulated) were systematically characterized.

### Experimental validation by RT-qPCR

To validate transcriptomic results, five DEGs (*Mx1, IRF3, LOC101822825, DPF3*, and *CD244*) were randomly selected for RT-qPCR analysis. Gene-specific primers were designed for each target, and amplification was performed using SYBR Green chemistry. GAPDH was used as an endogenous control for normalization, and relative gene expression was calculated using the 2^-ΔΔCt^ method.

## Results

### ^12^C^6+^ heavy-ion mutagenesis screening yields BHK-21 clones with stable, bipolarized FMDV replication phenotypes

**Figure 2:**
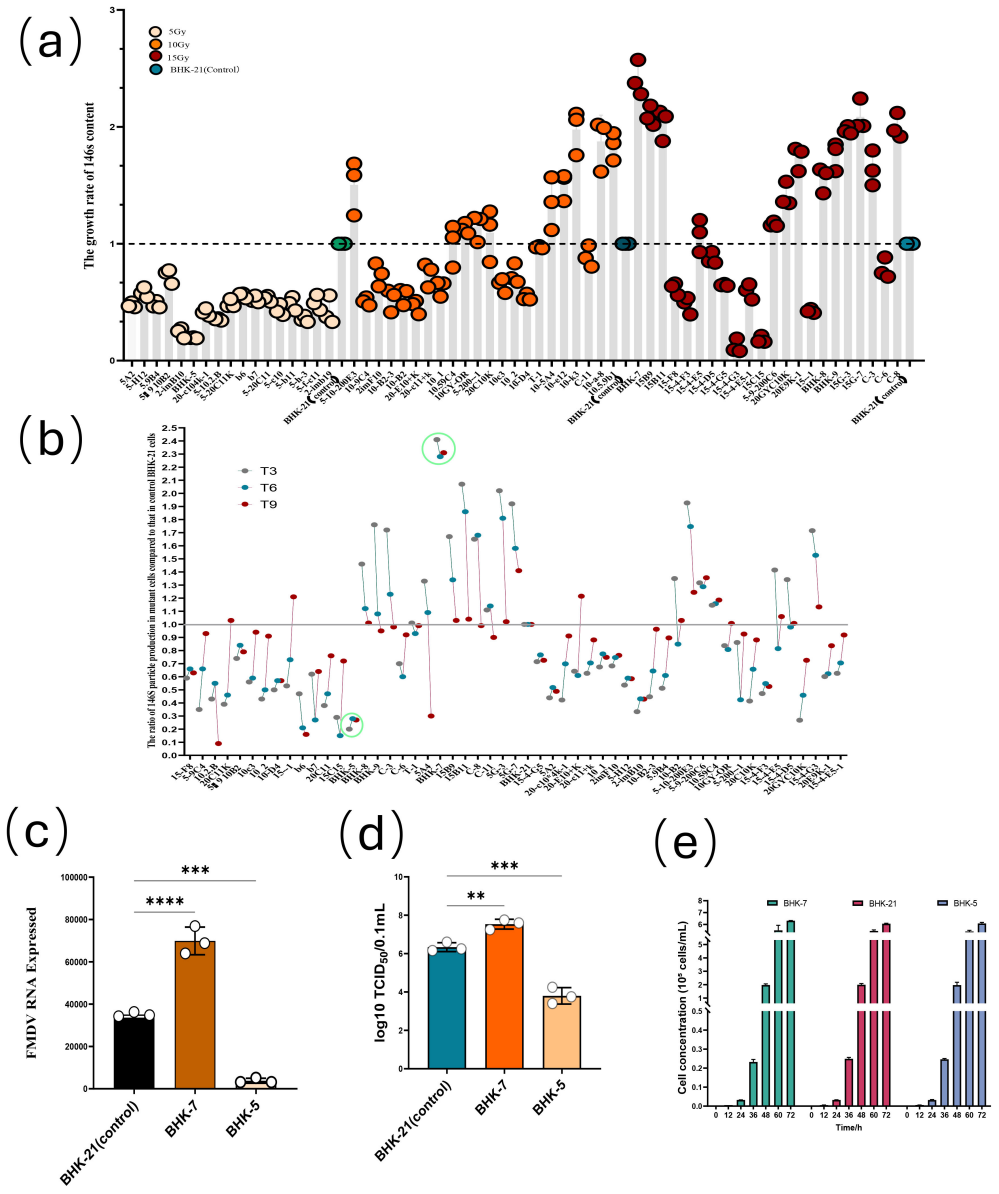
Phenotypic characterization and stability of FMDV replication in monoclonal BHK-21 cell lines following ^12^C^6+^ irradiation screening. **(a)** Initial screening of 63 monoclonal cell lines based on the ratio of FMDV 146S antigen content in each clone relative to parental BHK-21 cells post-infection. Clones are categorized by irradiation dose: 5 Gy (beige), 10 Gy (orange), and 15 Gy (dark red). Parental BHK-21 controls are shown as blue-outlined bars. **(b)** Stability of the viral replication phenotype across successive passages in 52 viable clones, presented as the 146S antigen ratio (χ) relative to control cells. Clonal stability was determined by monitoring the fluctuation of χ across three key passages (T3, T6, T9). A stable phenotype was defined by fluctuations within a ±0.2 range, with χ values progressively stabilizing close to 1. BHK-5 and BHK-7 (highlighted with green circles) were selected as stable anti-viral and pro-viral clones, respectively. **(c)** Relative FMDV RNA expression levels in the selected anti-viral (BHK-5) and pro-viral (BHK-7) clones compared to parental BHK-21 cells. **(d)** Viral titers (expressed as log_10_ TCID_50_/0.1 mL) produced by BHK-5, BHK-7, and parental BHK-21 cell lines. **(e)** Growth kinetics of BHK-5, BHK-7, and parental BHK-21 cell lines, demonstrating comparable proliferation rates across all groups. Data in panels **(c–e)** are presented as the mean ± standard deviation (SD) from three independent experiments. Statistical significance was determined using Student’s t-test: *p < 0.05, ***p < 0.001, ****p < 0.0001.

We previously established a series of mutant clones through heavy ion irradiation (Song et al., 2025a; Song et al., 2025b). In this study, we conducted a detailed phenotypic screening and stability analysis on these clones. Parental BHK-21 cells were irradiated with ^12^C^6+^heavy ions (5, 10, 15 Gy) to generate cell lines with stable, divergent viral replication phenotypes. From an initial pool of 63 monoclonal cell lines, we employed a multi-step screening process to identify clones exhibiting stable, heritable phenotypes (**Figure 2**). A primary screen based on FMDV 146S antigen content revealed a striking dose-dependent polarization in viral replication capacity (**Figure 2A**). Clones from the low-dose (5 Gy) group predominantly restricted FMDV replication, whereas those from the high-dose (15 Gy) group were largely highly pro-viral and supported high viral yields. This observation suggests that the intensity of mutagenic stress may steer cells toward distinct phenotypic trajectories.

Following rigorous passaging and stability analysis, which eliminated unstable clones, two lines with consistent, opposing phenotypes were selected for in-depth characterization (**Figure 2B**). The stability of the viral replication phenotype was critically assessed by infecting mutant and control parental BHK-21 cells at identical densities with FMDV and measuring the ratio of 146S antigen production. A stable phenotype was defined as a consistent ratio (either high or low) across three key passages (T3, T6, T9). Clones exhibiting significant fluctuation in this ratio or a gradual convergence towards a ratio of 1 (indicating a return to the parental BHK-21 level of 146S production) were deemed genetically unstable or prone to phenotypic reversion; such clones were excluded from further study. The BHK-5-line (from the 5 Gy group), which consistently exhibited the lowest viral yield, was designated as the representative anti-viral clone. Conversely, the BHK-7-line (from the 15 Gy group), which maintained the highest viral yield, was designated as the representative pro-viral clone.

This pronounced bipolarization was further confirmed by assessing viral nucleic acid synthesis and infectious particle production. RT-qPCR analysis revealed markedly suppressed viral mRNA expression in BHK-5 cells, but significantly enhanced expression in BHK-7 cells, compared to the parental line (**Figure 2C**). Similarly, viral titer measurements (TCID_50_) showed that BHK-5 produced dramatically less infectious virus, while BHK-7 produced substantially more (**Figure 2D**). Most importantly, growth curve analysis confirmed comparable proliferation rates among all three cell lines (**Figure 2E**), ruling out differential cell growth as a confounding factor and establishing that the divergent viral yields originate from intrinsic differences in host–virus interactions (**Table 1**).

**Table 1:** Comparison of phenotypic and molecular characteristics of parental, anti-viral (BHK-5), and pro-viral (BHK-7) cell lines.

Feature	BHK-21	Anti-viral clone BHK-5	Pro-viral clone BHK-7
Phenotype	Intermediate	Antiviral/Anti-viral	Proviral/Pro-viral
Source Dose	N/A	5 Gy	15 Gy
Proliferation Rate	Normal	Comparable to Parental	Comparable to Parental
FMDV 146S Yield	Baseline	Significantly Decreased	Significantly Increased
FMDV Titer (TCID_50_)	Baseline	Drastically Reduced	Substantially Increased
Ultrastructure (post-infection)	Vacuolization, Autophagy	Apoptotic Blebbing, Rapid Destruction	Virus Factories,Cytoplasmic Integrity
Baseline Immune Status	Quiescent	Pre-activated:*RIG-I* & *TLR* Pathways Upregulated	Suppressed: *TNF* Signaling Pathway Downregulated
Response to FMDV	Moderate IFN Response	Rapid & Robust: Strong *IFN* Signaling, Apoptosis	Evasive: Blunted IFN Response,*G2/M* Cell Cycle Hijacking
Metabolic Profile	Baseline	Suppressed (e.g., *P450*)	Enhanced: Glycolysis & *PI3K-Akt* Pathways Upregulated
Key Upregulated TF Families	N/A	IRF Family	*Runt* Family,*MYC_N, Homeobox*
Key Reprogrammed TF Families	N/A	*zf-C2H2* Altered	*zf-C2H2* Significantly Altered

### Bipolarized phenotypes are driven by global transcriptomic reprogramming, culminating in divergent ultrastructural fates

To elucidate the molecular basis of the opposing phenotypes, we performed global transcriptome analysis. Differential gene expression analysis revealed that heavy-ion mutagenesis induced massive, stable reprogramming of the cellular transcriptome—even in the absence of infection. Compared to parental BHK-21 cells, uninfected BHK-5 and BHK-7 lines each exhibited thousands of DEGs, with this number further increasing upon FMDV infection (**Table 2**).

**Table 2:** Differential gene expression statistics.

Comparison group	Down-regulated genes	Up-regulated genes	Total DEGs
BHK-5vsBHK-21	1305	1461	2766
(BHK-7 + FMDV)vs (BHK-21 + FMDV)	1554	2251	3805
BHK-7vsBHK-21	1202	1881	3083
(BHK-5 + FMDV)vs (BHK-21 + FMDV)	1331	2090	3421

**Figure 3:**
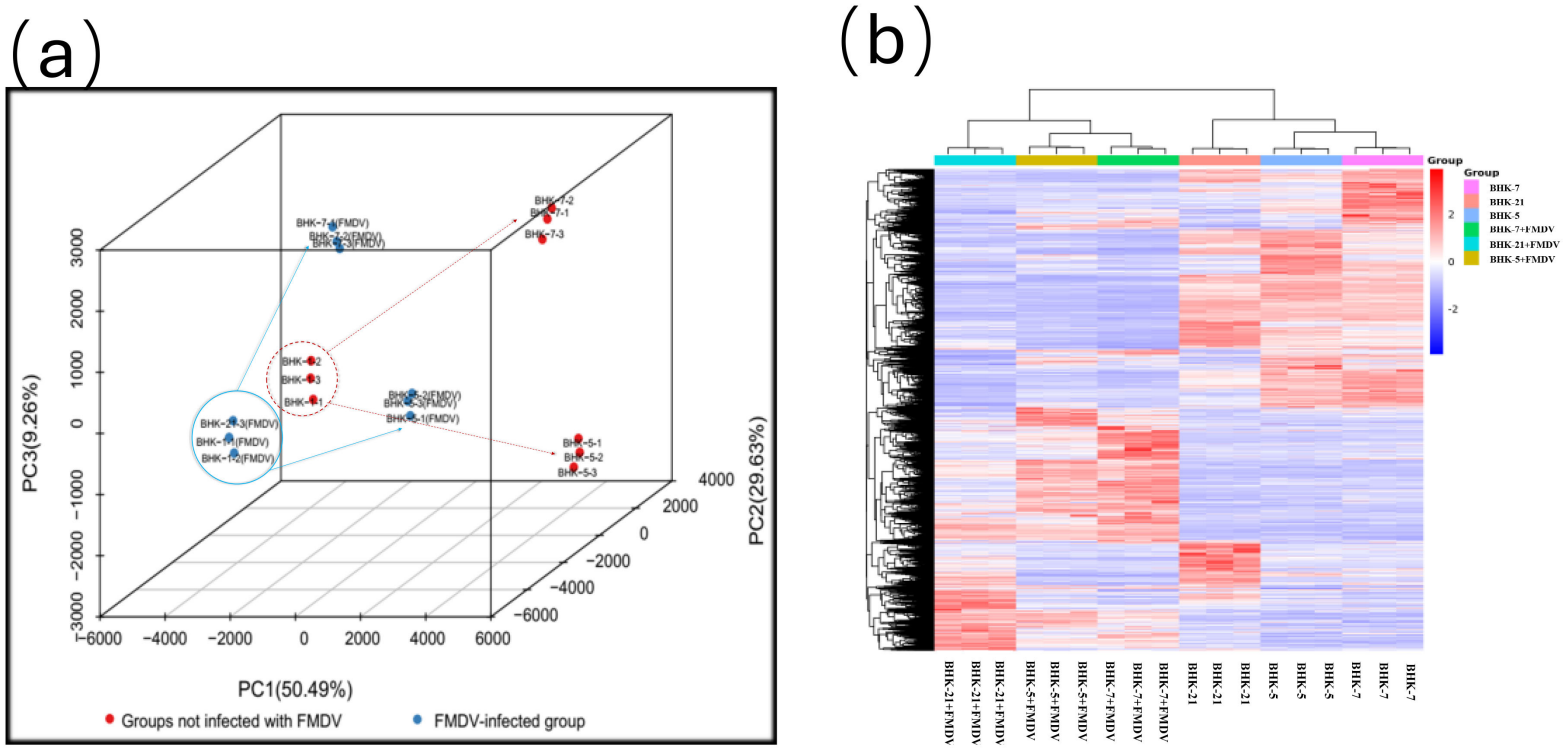
Transcriptomic divergence among BHK-5, BHK-7, and parental BHK-21 cells before and after FMDV infection. **(a)** Three-dimensional principal component analysis (PCA) of global gene expression profiles in BHK-5, BHK-7, and parental BHK-21 cells under uninfected and FMDV-infected (MOI = 0.1) conditions. **(b)** Hierarchical clustering heatmap of DEGs across all cell lines before and after infection with FMDV (MOI = 0.1).

**Figure 4:**
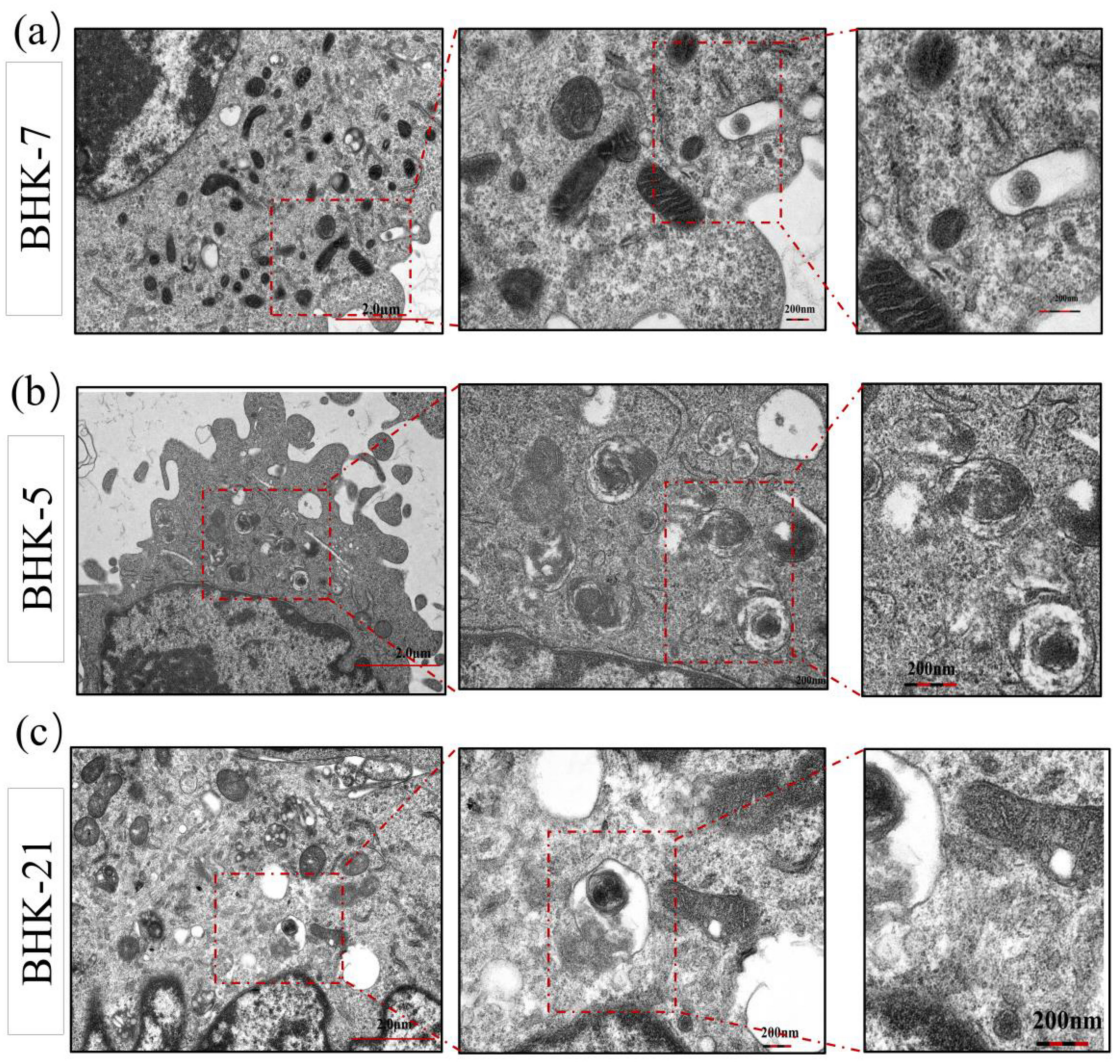
Ultrastructure of BHK lines at 6 h postinfection with FMDV. Representative transmission electron microscopy (TEM) images of ^12^C^6+^-mutagenized BHK-7 and BHK-5 cells, and parental BHK-21 cells at 6 hours post-infection (hpi) with FMDV. **(a)** BHK-7 cells (pro-viral phenotype) exhibit extensive viral replication complexes and abundant mature virions, while maintaining overall cellular integrity. This morphology correlates with molecular mechanisms supporting viral immune evasion and metabolic exploitation by the virus. **(b)** BHK-5 cells (anti-viral phenotype) display severe cytopathic effects, including membrane blebbing indicative of apoptosis. This rapid cytopathic response aligns with enhanced interferon signaling and early cell death. **(c) **Parental BHK-21 cells show an intermediate response, marked by cytoplasmic vacuolation and entrapment of virions within double-membraned autophagosome-like structures—suggesting activation of autophagy-related defense mechanisms.

Principal component analysis (PCA) of global expression profiles visualized this profound divergence (**Figure 3A**). In the uninfected state, BHK-5, BHK-7, and parental cells segregated into three distinct clusters, confirming that mutagenesis had established fundamentally divergent baseline transcriptomic profiles. FMDV infection functioned as a potent amplifier of this divergence, dramatically increasing the separation between clusters. Hierarchical clustering of all DEGs further corroborated this observation, revealing clear demarcation between infected and uninfected samples alongside a polarized “red-blue” expression pattern—characteristic of directional reprogramming toward either pro-viral or anti-viral states (**Figure 3B**). To link transcriptomic reprogramming to tangible biological outcomes, we examined the ultrastructural fate of cells post-infection via TEM (**Figure 4**). BHK-7 cells transformed into highly efficient “virus factories” (den Boon et al., 2024), with cytoplasm packed with viral replication complexes and mature virions, while maintaining overall cellular integrity. In stark contrast, BHK-5 cells underwent rapid, catastrophic destruction: their morphology was severely disrupted, with pronounced plasma membrane blebbing (a hallmark of apoptosis), suggesting a “scorched earth” strategy to limit viral spread consistent with functional enrichment analysis showing activation of interferon-driven apoptotic programs. Parental BHK-21 cells displayed an intermediate phenotype, with cytoplasmic vacuolation and evidence of autophagy, These findings highlight how mutagenesis drives daughter clones toward two distinct functional extremes. Collectively, the data establishes a clear association: profound and stable transcriptomic reprogramming underlies the divergent, bipolar cell fates following viral challenge.

### Functional analysis reveals pre-configured anti-viral and pro-viral cellular states

**Figure 5:**
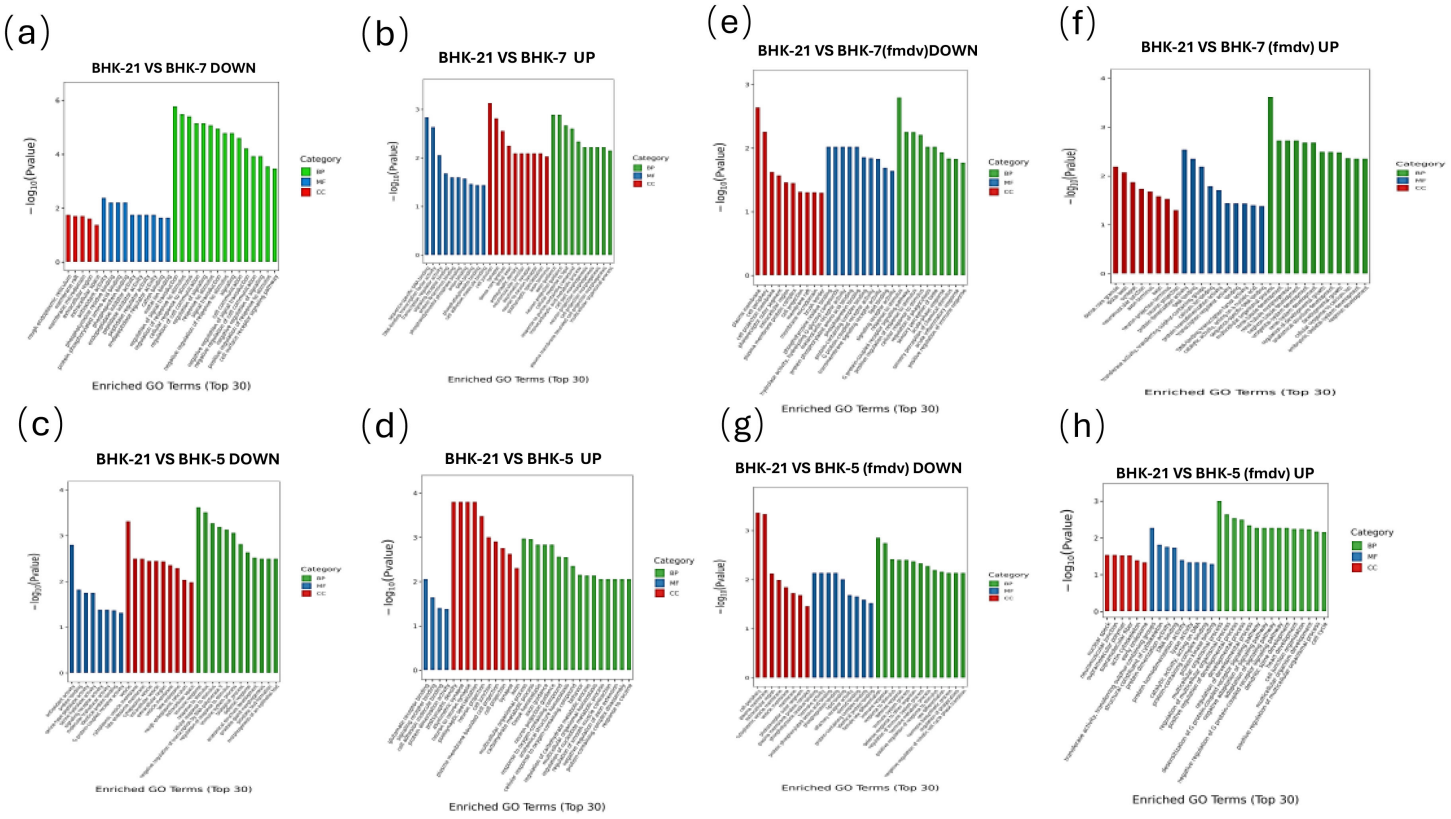
GO enrichment analysis of DEGs across BHK cell lines under uninfected and infected conditions. DEGs were functionally enriched across three GO categories: Biological Process (BP), Molecular Function (MF), and Cellular Component (CC). The x-axis indicates significantly enriched GO terms (Top 30); the y-axis represents the enrichment significance as -log10(Pvalue). Bar color corresponds to the GO category (BP, MF, or CC). Each panel shows a specific comparison between parental BHK-21 and derivative cell lines (BHK-5 or BHK-7) under uninfected or FMDV-infected (MOI = 0.1, 6 hpi) conditions: **(a)** BHK-21 vs. BHK-7, downregulated (uninfected); **(b)** BHK-21 vs. BHK-7, upregulated (uninfected); **(c)** BHK-21 vs. BHK-5, downregulated (uninfected); **(d)** BHK-21 vs. BHK-5, upregulated (uninfected); **(e)** BHK-21 vs. BHK-7, downregulated (infected); **(f)** BHK-21 vs. BHK-7, upregulated (infected); **(g)** BHK-21 vs. BHK-5, downregulated (infected); **(h)** BHK-21 vs. BHK-5, upregulated (infected).

**Figure 6:**
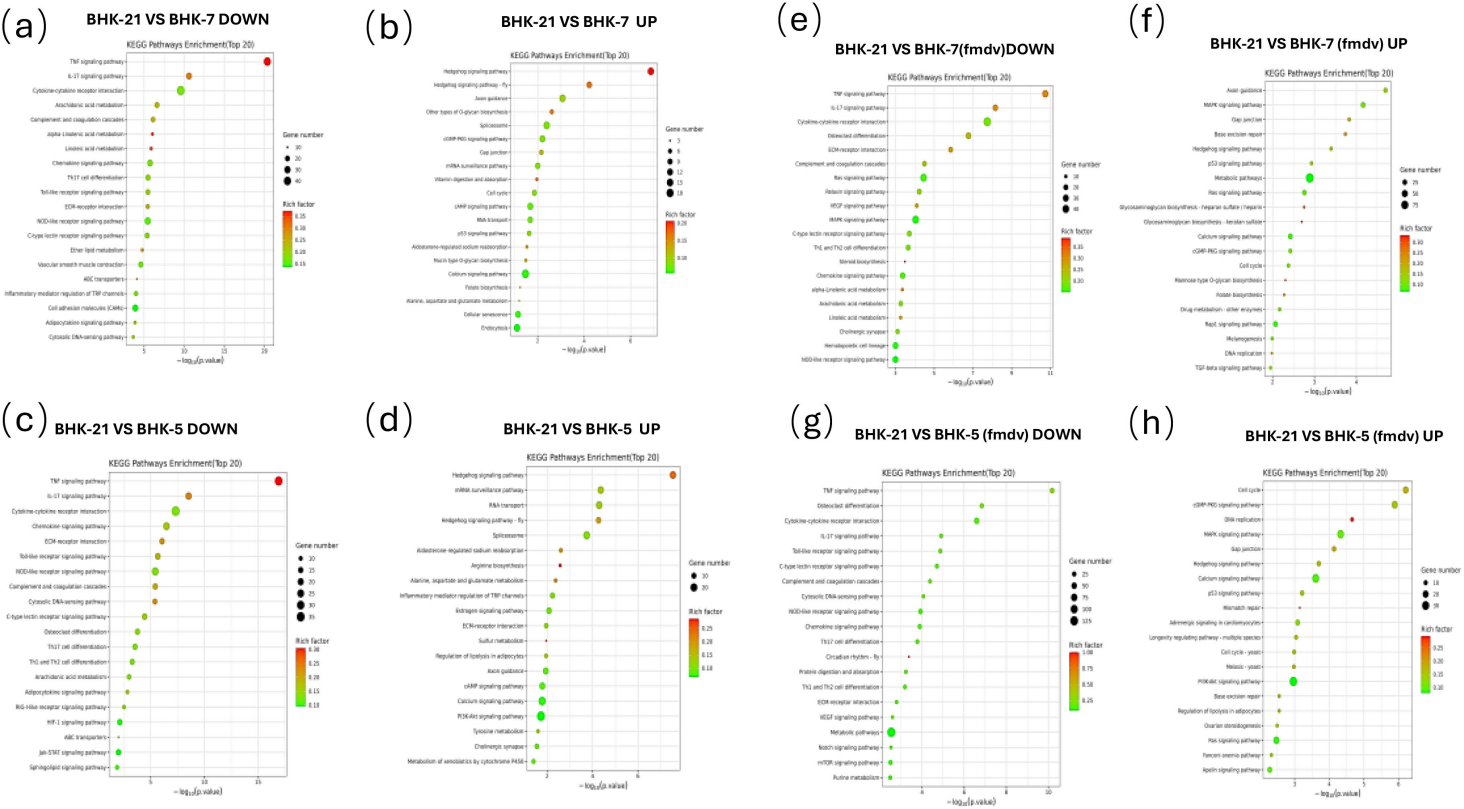
KEGG enrichment of DEGs across BHK cell lines under uninfected and FMDV-infected conditions. KEGG pathway enrichment was performed on DEGs identified via pairwise comparisons. The y-axis indicates significantly enriched pathways (Top 20); the x-axis represents the statistical significance as -log10(pvalue). Point size corresponds to the number of DEGs per pathway ("Gene number"), and color reflects the “Rich factor”. Each panel represents a specific comparison under uninfected or infected (MOI = 0.1, 6 hpi) conditions: **(a)** BHK-21 vs. BHK-7, downregulated (uninfected); **(b)** BHK-21 vs. BHK-7, upregulated (uninfected); **(c)** BHK-21 vs. BHK-5, downregulated (uninfected); **(d)** BHK-21 vs. BHK-5, upregulated (uninfected); **(e)** BHK-21 vs. BHK-7, downregulated (infected); **(f)** BHK-21 vs. BHK-7, upregulated (infected); **(g)** BHK-21 vs. BHK-5, downregulated (infected); **(h)** BHK-21 vs. BHK-5, upregulated (infected).

To interpret the functional consequences of transcriptomic reprogramming, we performed Gene Ontology (GO) and Kyoto Encyclopedia of Genes and Genomes (KEGG) pathway enrichment analyses (**Figures 5**, **6**). Results showed that the two clones were not merely different but pre-configured into functionally opposing states: an anti-viral “fortress” (BHK-5) and a pro-viral “factory” (BHK-7).

### BHK-5 clones are pre-set as an anti-viral “fortress”

Even before infection, BHK-5 cells existed in a state of heightened immune readiness. KEGG analysis showed that upregulated genes in uninfected BHK-5 were significantly enriched in the “*RIG-I-like* receptor signaling pathway” and “*Toll-like* receptor signaling pathway” (**Figure 6D**) the primary sensors of viral invasion. GO analysis corroborated this, with enrichment in “inflammatory response biological processes” and “receptor-mediated signaling molecular functions” (**Figure 5C**).

Upon FMDV infection, this pre-activated state translated into a swift, robust counterattack: upregulated genes became strongly enriched in downstream “interferon signaling” pathways (**Figure 6H**) and GO terms related to “viral defense response” and “apoptosis regulation” (**Figure 5G**)—consistent with induction of interferon-stimulated genes (ISGs) (Chin and Cresswell, 2001; Cui et al., 2025) and suppression of FMDV-mediated innate immune evasion (Li et al., 2023). This forms a coherent functional narrative: BHK-5 is pre-programmed to detect viruses and respond by triggering a potent interferon-driven apoptotic program—consistent with TEM observations of rapid cell death.

### BHK-7 clones are remodeled into a pro-viral “factory”

In stark contrast, BHK-7 cells were pre-programmed for robust biosynthesis and immune evasion. In the uninfected state, upregulated genes were enriched in “glycolysis/gluconeogenesis” and the “*PI3K-Akt* signaling pathway” (**Figure 6B**)—indicating a Warburg-like metabolic state ideal for supplying energy and building blocks for viral replication (Moreno-Altamirano et al., 2019; Camps et al., 2025). GO analysis supported this, with enrichment in “RNA processing” and “ribosome biogenesis” (**Figure 5B**)—suggesting the cell’s protein synthesis machinery was pre-primed for high-throughput production. Concurrently, BHK-7 exhibited pre-existing immune suppression, with significant downregulation of the critical “*TNF* signaling pathway” (**Figure 6A**).

Upon infection, this pro-viral state was consolidated: upregulated genes were enriched in pathways that hijack host functions, such as the “*G2/M* phase transition of the cell cycle” (Fu et al., 2024) a classic viral strategy to halt host processes and monopolize cellular resources. This functional profile fully explains BHK-7’s role as an efficient virus factory: it actively suppresses immunity while providing abundant metabolic and biosynthetic resources for viral amplification.

### Reprogramming of transcription factor networks is the central hub regulating bipolarized host responses

**Figure 7:**
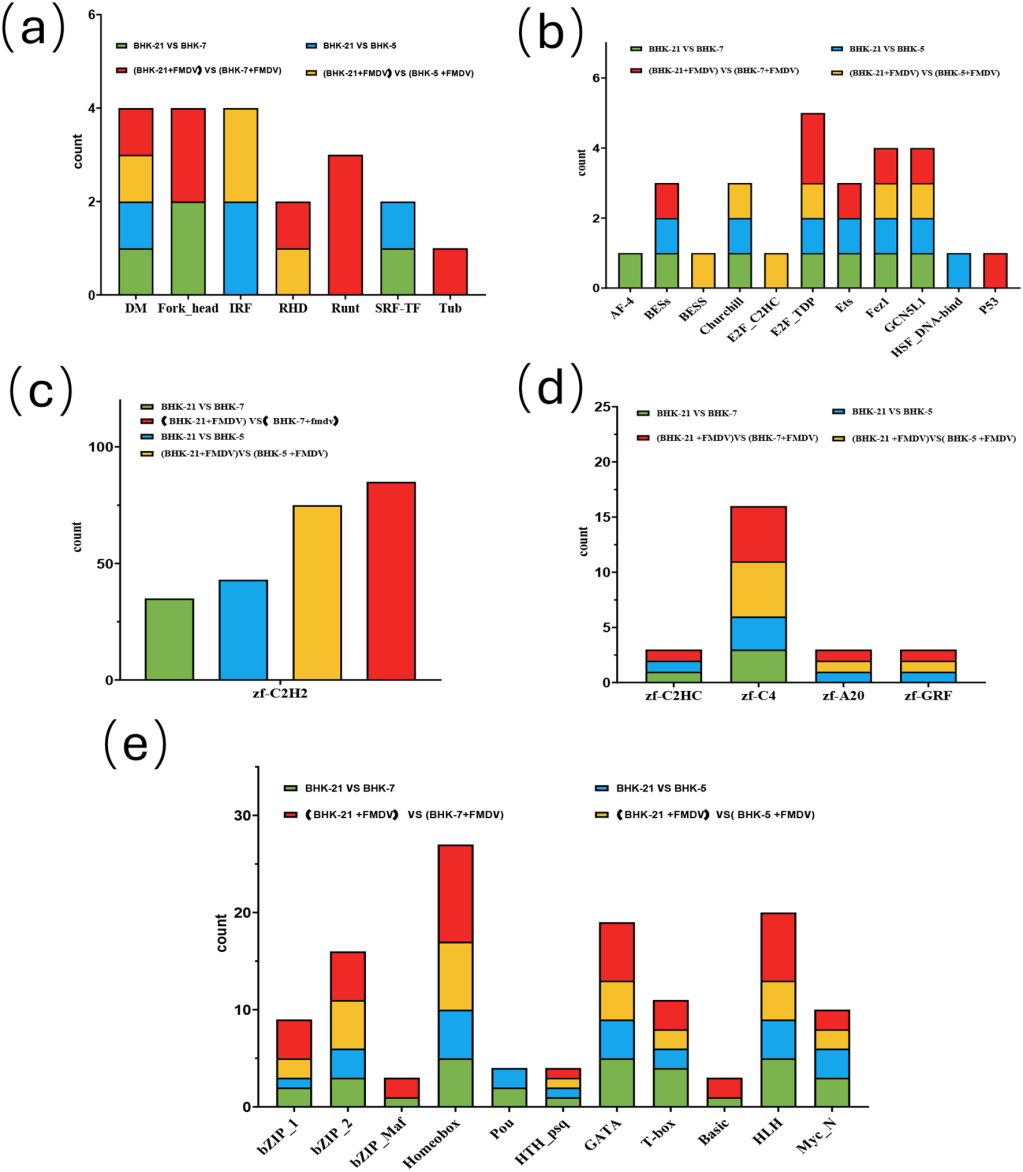
Statistics of differentially express transcription factor (TF) families. Bar charts show the number of differentially expressed TFs across various families in pairwise comparisons between parental BHK-21, anti-viral BHK-5, and pro-viral BHK-7 cells (with and without FMDV infection). **(a)** Key immune and developmental TFs; **(b)** TFs related to cell cycle and DNA damage; **(c)** The abundant zf-C2H2 family; **(d)** Other zinc-finger families; **(e)** TFs involved in development, metabolism, and signaling.

The profound coordinated changes in cellular function strongly suggested that the core “operating system” of the cells—their TF networks—had been fundamentally rewired. Analysis of differentially expressed TFs confirmed this, identifying master regulators driving bipolarization (**Figure 7**). The *zf-C2H2* zinc-finger family—the largest class of mammalian TFs—was profoundly altered (Kamaliyan and Clarke, 2024), implying fundamental reprogramming of the cell’s genomic defense posture (these TFs are key regulators of viral and endogenous retroelement silencing) (Lee et al., 2024). Beyond this global shift, specific TF families have been bipolarized.

The anti-viral BHK-5 clone was characterized by a strong interferon-response signature: the Interferon Regulatory Factor (*IRF*) family was uniquely and consistently enriched in BHK-5, particularly after FMDV infection (**Figure 7A**). In contrast, the pro-viral BHK-7 clone displayed a signature of immune evasion and developmental reprogramming: the Runt family of TFs—known for roles in immune suppression (Seo and Taniuchi, 2020) were specifically induced in infected BHK-7 cells (**Figure 7A**), consistent with their ability to bind viral genomes (Kim et al., 2020) and repress anti-viral responses (Lu et al., 2024). Additionally, TFs associated with cell-cycle control (p53), metabolic reprogramming (*MYC_N*), and developmental plasticity (*Homeobox, HLH*) were most prominent in infected BHK-7 cells (**Figures 7B, E**). These divergent TF landscapes demonstrate that heavy-ion mutagenesis reconfigured master regulatory circuits, predisposing BHK-5 to aggressive immune responses and BHK-7 to immune tolerance and metabolic support for viruses.

### Integrative network analysis reveals antagonistic regulation of key functional modules

**Figure 8:**
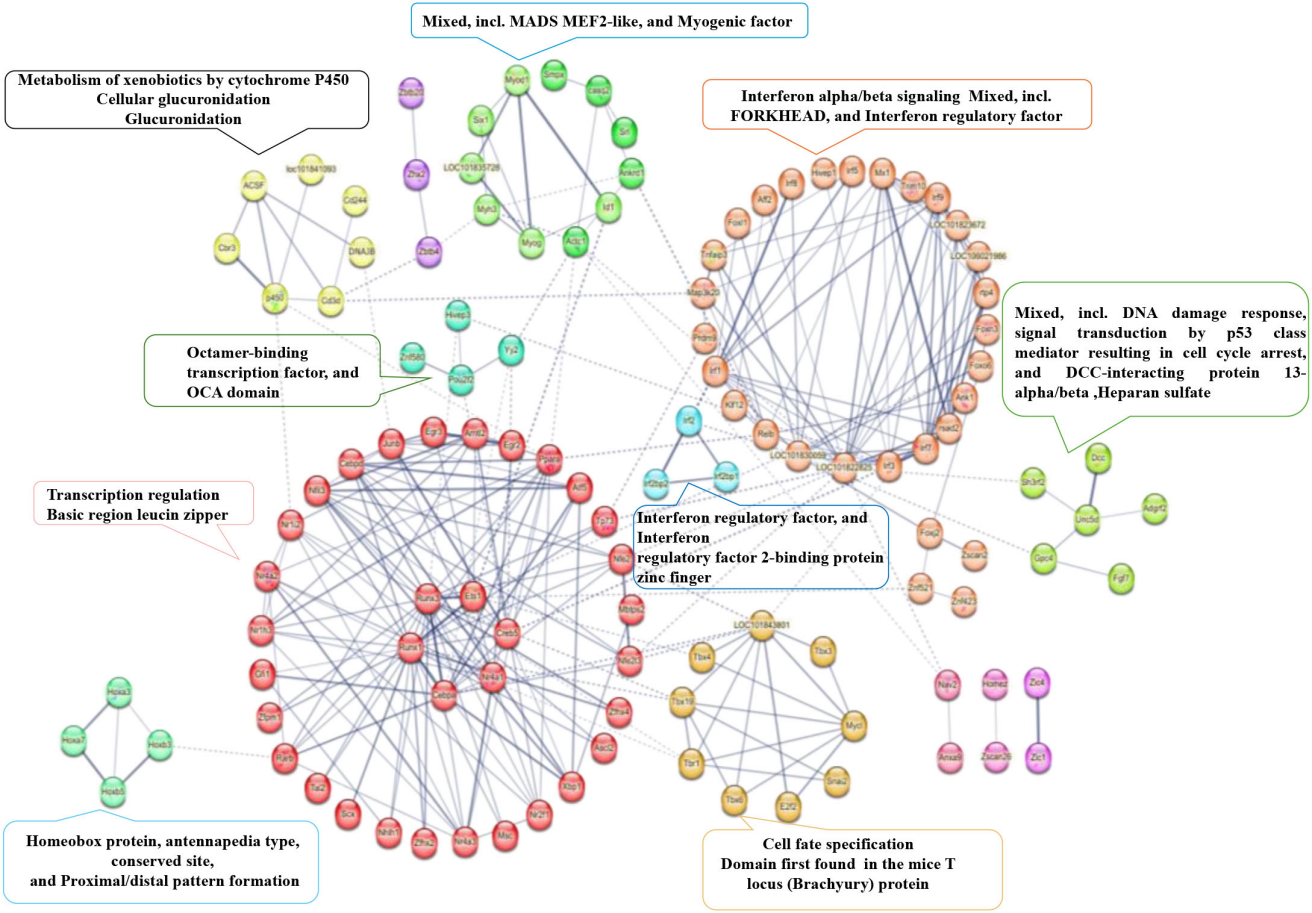
Protein–protein interaction (PPI) network of DEGs. The network illustrates functional modules enriched among DEGs. Nodes represent proteins (genes), and edges represent known interactions. Major functional clusters are highlighted and annotated: interferon signaling (orange), DNA damage response/*p53* signaling (blue), metabolism (yellow), and myogenesis (green).

**Figure 9:**
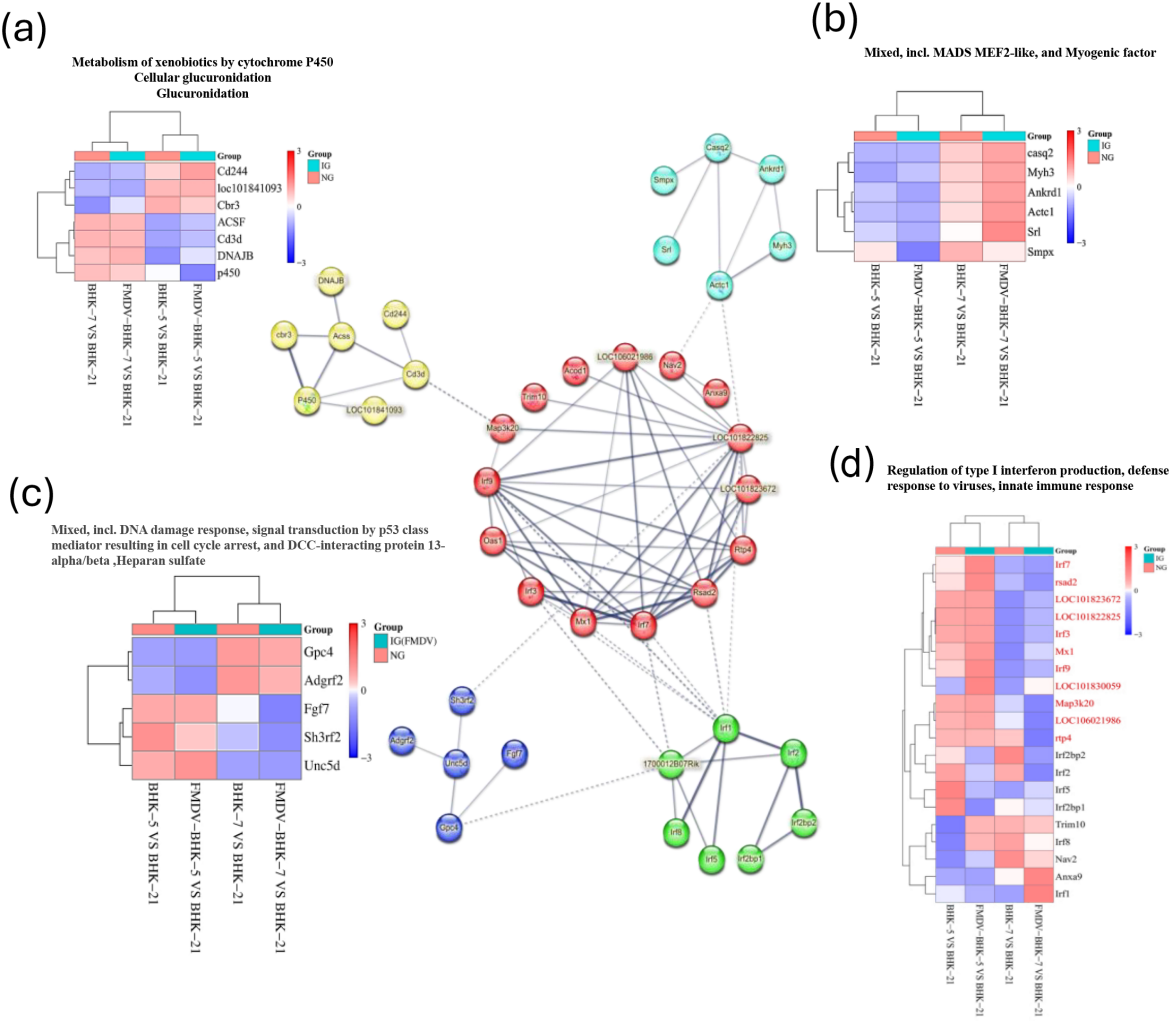
Integrated analysis of functional modules and gene expression. The core PPI network is shown in the center. Surrounding heatmaps correspond to key functional modules, displaying gene expression changes across four comparisons: BHK-7 vs. BHK-21 (uninfected), BHK-7 vs. BHK-21 (infected), BHK-5 vs. BHK-21 (uninfected), and BHK-5 vs. BHK-21 (infected). Red indicates upregulation; blue indicates downregulation. **(a)** Metabolism of xenobiotics by cytochrome P450; **(b)** Myogenic factors; **(c)** DNA damage response and p53 signaling; **(d)** Type I interferon production and defense response to virus.

To visualize how rewired TF networks translate into functional outcomes, we constructed a protein-protein interaction (PPI) network from DEGs using the STRING database. The analysis was performed against the *Mesocricetus auratus* (golden hamster) proteome background. We integrated interaction evidence from multiple sources, including experimental data, databases, and co-expression. The resulting network, which encompasses both physical and functional associations, was visualized and initially analyzed using the built-in tools in STRING’s interactive interface before further biological interpretation (**Figure 8**). Analysis showed that DEGs clustered into distinct functional modules—including interferon signaling, metabolism, DNA damage response, and myogenesis—providing a blueprint of key cellular processes under antagonistic regulation. Integrating this network with expression data directly visualized opposing regulatory programs in BHK-5 and BHK-7 (**Figure 9**), revealing three major antagonistically controlled regulatory axes:

The IFN–IRF Anti-Viral Axis: This axis represents the core anti-viral response. In infected BHK-5 cells, the entire interferon response module was massively, coordinately upregulated, including key TFs (*Irf7*, Stat1, Stat2) and potent anti-viral effector genes (e.g.,*Rsad2*,*Mx1*, multiple *fit* family members; **Figure 9D**) consistent with induction of *IFN*-driven anti-viral programs (Chin and Cresswell, 2001; Li et al., 2023; Cui et al., 2025). This reflects rapid mobilization of a powerful anti-viral state. In stark contrast, this module was unresponsive or downregulated in infected BHK-7 cells, providing clear evidence of immune evasion.

The Metabolism–Structural Remodeling Axis: The two clones exhibited opposing metabolic and structural strategies. BHK-5 cells suppressed key metabolic pathways, with significant downregulation of genes involved in xenobiotic metabolism by cytochrome *P450* (**Figure 9A**) effectively starving the virus of resources (Moreno-Altamirano et al., 2019; Camps et al., 2025). Conversely, BHK-7 cells sustained metabolic activity and initiated a notable structural remodeling program, evidenced by robust, specific upregulation of myogenic factors and muscle-related genes (e.g., *Casq2*, *Actc1*; **Figure 9B**). Notably, upregulation of myogenic factors in infected BHK-7 cells suggests profound cytoskeletal remodeling to support viral replication complex formation—consistent with known mechanisms of picornavirus cellular remodeling (Bouin and Semler, 2020; den Boon et al., 2024).

The DNA Damage and Cell Fate Axis: Both cell lines showed alterations in genes related to DNA damage response and cell-cycle control, mediated by the *p53* pathway (**Figure 9C**). Although *p53* signaling was activated in both clones, the specific expression patterns of downstream effector genes diverged—such as the upregulation of *Gpc4* in BHK-7—implying that the initial mutagenic event induced long-term reprogramming of cell fate and stress response pathways (Kumar et al., 2023; Tong et al., 2024). These differential expression patterns likely steered the cells toward distinct outcomes: BHK-5 cells tended toward apoptosis, whereas BHK-7 cells may have undergone cell cycle arrest, a state potentially favoring viral replication.

### A systems-level model of mutagenesis-driven bipolarization of viral replication and its validation

**Figure 10:**
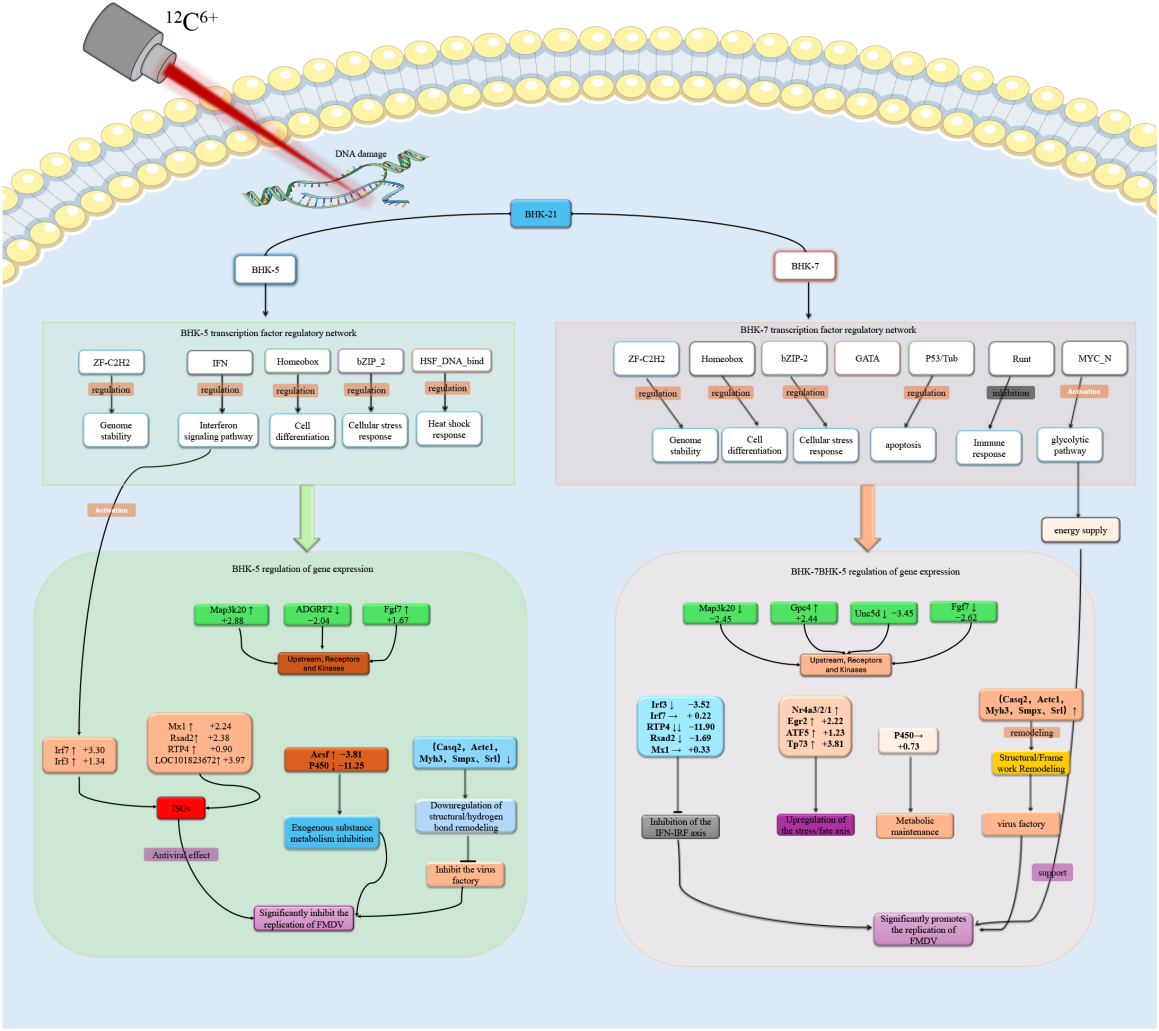
Induction of polarized regulatory networks by heavy-ion mutagenesis in BHK cells upon FMDV infection. The diagram illustrates divergent molecular mechanisms underlying the anti-viral clone BHK-5 (left, green pathway) and pro-viral clone BHK-7 (right, gray pathway). Top: The initial irradiation event (5 Gy vs. 15 Gy) leads to distinct outcomes: DNA damage response (DDR)-induced immune pre-activation vs. selection for immune tolerance. This establishes a pre-configured TF network layer: BHK-5 is enriched in immune-activating IRFs, while BHK-7 is enriched in immunosuppressive Runt and metabolic *MYC_N* families. Bottom: The functional module layer shows BHK-5 activating the *IFN–IRF* axis (*Map3k20, Irf3, Irf7*) and *ISGs* (*Mx1, Rsad2*) while suppressing metabolism (P450, Acsf). BHK-7 suppresses the *IFN–IRF* axis (*Irf3, RTP4*), activates stress/metabolic pathways, and remodels cells to promote efficient FMDV replication.

**Figure 11:**
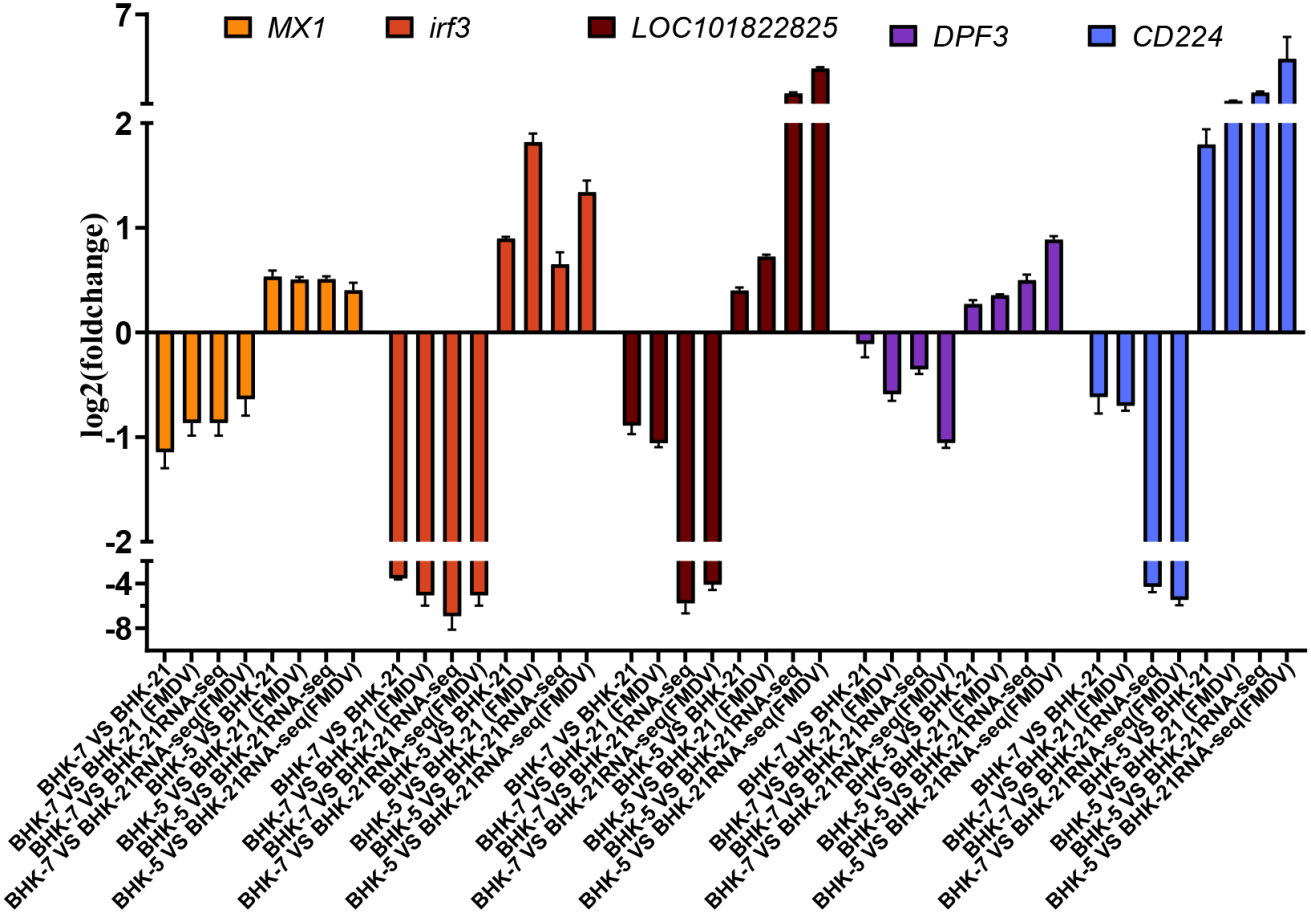
Validation of DEGs by RT-qPCR. The relative expression fold change of five selected genes in BHK-7 and BHK-5 cells (before and after FMDV infection) was measured by RT-qPCR. Results are consistent with RNA-seq data, confirming the reliability of transcriptomic findings.

To integrate these multi-layered findings, we developed a comprehensive systems-level model illustrating how heavy-ion mutagenesis drives the polarization of antiviral outcomes in BHK-5 and BHK-7 clones (**Figure 10**). The model delineates the entire cascade: from initial DNA damage and resultant stable genomic alterations— which, in turn, trigger either adaptive immune pre-activation (Hanot et al., 2012; Kumar et al., 2023; Chailapakul et al., 2024) or selection of immune-tolerant survivors— through the consequent stable rewiring of transcription factor (TF) networks, to the activation of divergent effector pathways, ultimately culminating in viral restriction or a pro-viral state. In the antiviral pathway of BHK-5 (left), the stably altered genomic landscape—manifested as a rewired TF network—primes cells for antiviral readiness. Upon infection, this configuration drives robust activation of type I interferon signaling via *IRF3*, upregulation of key interferon-stimulated genes (*ISGs; Mx1, Rsad2*) (Chin and Cresswell, 2001; Li et al., 2023; Cui et al., 2025), and concurrent suppression of virus-required metabolic pathways (*P450, ACSF*) (Moreno-Altamirano et al., 2019; Camps et al., 2025). This dual strategy of immune amplification and metabolic starvation creates a hostile microenvironment that abrogates FMDV replication. In the pro-viral pathway of BHK-7 (right), genomic changes promote a TF network configuration enabling immune evasion and metabolic hijacking. The interferon response is impaired (downregulation of *Irf3* and *Rtp4*), while immune silencing is mediated by factors such as Runt (Kim et al., 2020; Seo and Taniuchi, 2020; Lu et al., 2024). This is coupled with sustained glycolysis and metabolic support (*P450*), which provides abundant resources for viral replication and renders BHK-7 a highly productive host (Bouin and Semler, 2020; den Boon et al., 2024). To validate the transcriptomic data, we selected five key DEGs (*MX1, Irf3, LOC101822825, DPF3, CD244*) for RT-qPCR analysis (**Figure 11**). Relative expression changes of these genes across all conditions were highly consistent with RNA-seq results, confirming the accuracy of our transcriptomic profiling and providing robust experimental support for the proposed model.

## Discussion

By employing high-linear energy transfer (high-LET) ^12^C^6+^ heavy-ion irradiation, we established two stable BHK-21-derived clones with diametrically opposed foot-and-mouth disease virus (FMDV) susceptibilities: BHK-7 (markedly enhanced viral replication) and BHK-5 (highly antiviral to infection). *Our findings suggest* that the unique, complex DNA damage induced by heavy ions triggers profound, heritable reprogramming of the host transcriptional landscape—an outcome difficult to achieve with conventional mutagens (Yanagisawa et al., 2022; Guo et al., 2024; Ishii et al., 2024).

### The Dose-Dependent Paradox: An Induction vs. Selection Model

A central finding is the dose-dependent polarization of phenotypes: low-dose (5 Gy) irradiation predominantly yielded antiviral clones, whereas high-dose (15 Gy) irradiation produced proviral ones. This apparent paradox can be explained by a dual-mechanism “induction vs. selection” model. High-LET radiation elicits qualitatively distinct biological responses at low versus high doses (Chailapakul et al., 2024; Song et al., 2025b). The 5 Gy dose likely acts as a potent inducer of adaptive responses—analogous to radiation hormesis—where low-level stress activates cellular defense and repair systems. This “trains” surviving cells, pushing them into a stable, preactivated antiviral state (as seen in BHK-5). In contrast, the 15 Gy dose is highly cytotoxic (Kumar et al., 2023), imposing extreme selective pressure that eliminates most cells. Rare survivors likely harbor preexisting defects in apoptosis or immune signaling—defects that may be exacerbated by glutathione depletion (which potentiates heavy-ion-induced clustered DNA lesions) (Hanot et al., 2012). While these defects enable survival under massive DNA damage, they coincidentally render cells ideal, immune-tolerant hosts for viral replication—giving rise to the BHK-7 phenotype.

### From DNA Damage to Stable Phenotype: The Role of the DDR–cGAS–STING Axis

A critical question is how a transient physical insult (irradiation) induces stable, heritable changes in the cellular “operating system.” We propose that complex, clustered DNA double-strand breaks (DSBs) generated by high-LET heavy ions are the initiating trigger (Sutherland et al., 2000; Hagiwara et al., 2019; Mladenova et al., 2022a; Mladenova et al., 2022b). These severe lesions activate a robust DNA damage response (DDR), which is intricately linked to innate immunity (Tong et al., 2024). We hypothesize that repair of complex DNA damage leads to micronucleus formation or release of DNA fragments into the cytoplasm. This self-DNA is recognized as a danger signal by the cGAS–STING pathway—a primary driver of type I interferon (IFN) production. This initial DDR-driven immune activation may then establish long-term “epigenetic memory” or “trained immunity” via chromatin remodeling at key immune gene loci. This model provides a direct mechanistic link between initial DNA damage and the preactivated immune state observed in uninfected BHK-5 cells.

### Beyond Epigenetic and Transcriptional Reprogramming: Genomic Scars as a Foundation for Phenotypic Stability

Beyond epigenetic and transcriptional reprogramming, a fundamental mechanism driving the observed stable phenotypes may reside in the permanent genomic alterations elicited by high-LET radiation. A defining hallmark of heavy-ion irradiation is its capacity to induce complex, clustered DSBs. The repair of such severe lesions—particularly via error-prone pathways—can give rise to stable genomic structural variations (SVs), including microdeletions, inversions, and translocations (Goodarzi et al., 2008; Averbeck and Rodriguez-Lafrasse, 2021). We hypothesize that these SVs serve as a critical and enduring molecular foundation for clonal phenotypic divergence. For instance, a microdeletion within the promoter region of a key immune suppressive factor could result in its permanent silencing, thereby locking the BHK-5 clone into a state of constitutive antiviral pathway activation. Conversely, in BHK-7 cells, SVs disrupting the coding sequence or regulatory elements of a critical innate immune sensor might permanently impair the antiviral response, fostering a proviral cellular state. This model posits that these radiation-induced genomic scars—distinct from yet potentially synergistic with epigenetic memory—provide a robust, heritable basis for the diametrically opposed and stable transcriptional programs governing FMDV susceptibility.

### Master Regulators: Causal Rewiring of Transcription Factor Networks

Divergent phenotypes are ultimately executed by rewired transcription factor (TF) networks. The profound alteration of the zf-C2H2 zinc-finger family (Kamaliyan and Clarke, 2024) implies fundamental reprogramming of the cell’s genomic defense posture (these TFs are key to silencing viral and endogenous retroelements (Lee et al., 2024)). Beyond this global shift, specific TF families drive bipolarization. In the immune-suppressed BHK-7 clone, upregulation of Runt family TFs (Seo and Taniuchi, 2020) is particularly notable: this likely reflects activation of a conserved proviral host program, as RUNX TFs are exploited by diverse viruses to suppress host immunity (Kim et al., 2020) and promote replication or latency (Lu et al., 2024). Thus, 15 Gy selective pressure likely favored clones capable of activating this specific immune evasion pathway. This contrasts sharply with BHK-5, where IRF family enrichment executes the IFN-driven antiviral program (Chin and Cresswell, 2001; Li et al., 2023; Cui et al., 2025). These findings reframe TF changes not as mere phenotypic correlations, but as causal drivers of two distinct, preprogrammed cellular strategies.

### Deconstructing the “Virus Factory”: Myogenesis and Cytoskeletal Hijacking

Our analysis uncovered striking, specific upregulation of myogenesis-related genes in the proviral BHK-7 clone—providing a molecular explanation for the “virus factories” observed via transmission electron microscopy (TEM) (den Boon et al., 2024). Picornaviruses (including FMDV) extensively remodel host cell architecture—particularly the actin cytoskeleton—to construct specialized “replication organelles” for viral genome replication (Bouin and Semler, 2020). Myogenesis is a fundamental biological process centered on dramatic actin cytoskeleton reorganization to form muscle fibers. We propose that upregulation of the myogenesis program in BHK-7 provides the structural “raw materials” for building these virus factories. In this model, FMDV hijacks a host developmental pathway to physically reengineer cells for maximal viral production. This links a key transcriptomic finding to a critical ultrastructural observation, aligning with established principles of picornavirus biology (Hagiwara et al., 2019; Bouin and Semler, 2020).

### Implications and Future Directions

This study has both practical and fundamental implications. BHK-7—with its high viral yield—represents a promising substrate for industrial-scale inactivated FMD vaccine production (Berryman et al., 2025), offering potential improvements in antigen output, cost-effectiveness, and scalability. It complements existing advances in suspension culture (Park et al., 2021) and cell density optimization (Rizvi et al., 2022). Conversely, BHK-5 provides a robust model for delineating host restriction mechanisms, enabling precise genetic dissection of antiviral pathways (Li et al., 2023; Ma et al., 2025). Previous work on heavy-ion-irradiated BHK-21 cells supports the durability of phenotypic reprogramming (Song et al., 2025a) and informs optimal dosing and stabilization strategies (Yanagisawa et al., 2022; Guo et al., 2024). Our findings shift the focus of FMDV–host interaction research from viral factors alone to the preexisting cellular state as a key determinant of infection outcomes (Moreno-Altamirano et al., 2019; Camps et al., 2025).

Future studies should prioritize causal validation of identified TFs: CRISPR/Cas9-mediated knockout or overexpression of candidate TFs (e.g., Runt or zf-C2H2 family members) in parental BHK-21 cells, followed by quantitative assessment of FMDV replication, will test their necessity and sufficiency for conferring proviral or antiviral phenotypes. Integrating transcriptomic data with proteomic and metabolomic analyses will provide a more comprehensive understanding of reprogramming across immunity, metabolism, and cell-cycle regulation. Finally, long-term passaging and scaled-down bioreactor studies are essential to confirm genetic and phenotypic stability under industrial bioprocessing conditions.

## Conclusion

^12^C^6+^ heavy-ion mutagenesis is a powerful tool for inducing profound, stable reprogramming of host transcriptional networks. We generated a matched pair of BHK-21-derived cellular models that advance understanding of virus–host interactions and offer scalable substrates for improved FMD vaccine production.

## Data Availability

The datasets presented in this study can be found in online repositories. The names of the repository/repositories and accession number(s) can be found in the article/**Supplementary Material**.
